# Supplementary material for: The role of the arts in enhancing data literacy: A scoping review protocol
Source: PLoS One. 2023 Feb 13;18(2):e0281749. doi: 10.1371/journal.pone.0281749 (PMC9925065; doi:10.1371/journal.pone.0281749)
Supplement: Attachment — Submitted filename: Response to reviewers.docx [file pone.0281749.s002.docx]

**Response to reviewers**

We thank the reviewers for their positive and constructive feedback, which is particularly useful at this protocol stage of our project. We have addressed their comments on a point to point basis below.

Reviewer #1: *This submission outlines a scoping review protocol intended to synthesize academic understanding of how, when, and where the arts is being used to enhance data literacy. The methods are sound in relation to other reviews I have read or taken part in. The resulting review would be a valuable contribution to the growing body of work on arts and data literacy in multiple settings.*

**Response of the authors:** Thank you for the positive feedback on our review protocol.

*Introduction:
The review of rationale and context is clear and easy to understand for readers. The citations included are contextually appropriately. I have concerns about the scope being limited to academic products, because my personal experience in the field has shown numerous examples in k-12 learning settings that aren't producing formal academic outputs, and data art pieces in gallery settings. This is a well-known concern in mixed space reviews such as this one, so I encourage the authors to engage it and acknowledge it as a limitation to the methods.*

**Response of the authors:** We agree that this is a limitation of our review and have now added a sentence acknowledging this in the Discussion - ‘We acknowledge that this search is limited to academic products and may not capture all relevant outputs in this interdisciplinary space with the arts.’

*Materials and Methods
I am unfamiliar with scoping reviews, but they are summarized effectively for me. The queries in Table 3 needs some work - there are multiple examples of overlapping clauses (if I've understood the boolean syntax correctly). For instance, once "arts" is included there is no need to include "literary arts", "visual arts", etc. And "danc*" likely matches "dance" so that is redundant; same for "music*" and "musical instrument". In addition some clauses should be more generic, such as using "mosaic*" instead of "mosaics". These detailed comments should be taken in context of the search platform, whose syntax I am not an expert on (ie. does it auto-stem? match partial words? etc). I also might suggest including the phrase "performance", to capture the telling of a data story though arts means.*

**Response of the authors:** Thank you for the feedback on the search terms. We have included a new Table 3 with all terms of interest. We have also revised the search terms for the two databases in Table 4, removing overlapping terms and using wildcards where appropriate. We have added “performance art” as a search term. Other types of performance will be captured using the search terms e.g. story, arts, theatre. The preliminary searches have been re-run with the expanded date range (see response to reviewer 2) and the changes in search terms. The results are given in Table 4.

*Other comments:
The process will necessitate some hard-to-describe subjective judgements on inclusion/exclusion. This will make replication difficult, but the dual-review nature described should allow for transparency on application of the criteria.*

**Response of the authors:** We agree with the importance of transparency on applying the criteria and that the dual-review will help with this. We will also document reasons for exclusion during the process at the full-text screening stage.

Reviewer #2: *This manuscript outlines a protocol to guide a scoping review aiming to identify and map the available evidence for the role of the arts in enhancing data literacy. It is a very interesting topic and, in my opinion, the protocol is well conceived and proposed, deserving publication.*

**Response of the authors:** Thank you for the positive feedback on our review protocol.

*I have concerns on the abstract and the text (discussion), as follows:
Abstract: should indicate more precisely what has been done. It states “ to date, there has been no comprehensive review of publications on the role of the arts in the context of data literacy” and it follows… “The key aim of this proposed scoping review is to identify and map the available evidence for the role of the arts in enhancing data literacy.” The reader concludes that the paper will perform the scoping review. However, in the next sentence we discover that the paper proposes a protocol to perform the scoping review on this subject. The frustration of reading a paper with an expectation and not finding the final review should be mitigated with a starting statement in the abstract clearly showing its goal (my suggestion): “This paper presents a protocol and a search strategy framework to perform a scoping review to identify and map the available evidence for the role of the arts in enhancing data literacy”. PLOS ONE welcomes original research submissions from the natural sciences, medical research, engineering, as well as the related social sciences and humanities, including (…) Protocols (…) that describe detailed plans for research projects. It is the case in the present manuscript, that fits PLOS ONE scope.*

**Response of the authors:** We acknowledge that the abstract could have been clearer that this is a protocol and have now amended it to include the sentence suggested by the reviewer with the addition of methodological framework – “This paper presents a protocol and a methodological framework to perform a scoping review to identify and map the available evidence for the role of the arts in enhancing data literacy.”

*The text:
The authors adequately justify the need of the scoping review, present 4 Tables describing the concepts, definitions, and boundaries of the research question (Table 1), the eligibility criteria (Table 2), two examples of preliminary database searches (Table 3) showing 110 documents retrieved using the proposed strategy search in Scopus database, and 98 documents in Web of Science Core Collection, and preliminary charting elements and questions (Table 4) to apply in the documents found. The result is the proposed protocol as a methodological framework, which is interesting.*

**Response of the authors:** Thank you for the positive feedback on our methodological framework.

*Discussion could comment on some limitations of the study protocol. I can indicate at least 3:
(1) the use of a short ten-year period (>2012 publications), justified as “for currency”, but incompatible with the date of year of the Ottawa charter for health promotion (1986); the Fancourt and Finn scoping review published by WHO in 2019 relating the role of the arts and health cited in ref #9 presents 962 references, and the choice of starting this new scoping review at 2012 is not obvious nor necessarily adequate.*

**Response of the authors:** We acknowledge that the starting point for the review (2012) is arbitrary. We have now extended our search from 2002 to date (20 years) and have justified this by the use of relatively recent used of the term ‘data literacy’ which is rarely used before 2002 (we have found no references pre-2002 in preliminary searches). The preliminary searches have been re-run with the expanded date range and the changes in search terms. The results are given in Table 4.

*(2) the option for including only publications written in English: given that authors will search other sources that English-written databases (ERIC, Scholar google, grey literature, etc), it is a limitation to exclude, at least, French, Spanish or Portuguese written documents.*

**Response of the authors:** we acknowledge English language only is a limitation of this review (constrained by the first language of the authors) and have now acknowledged this in the discussion.

*(3) the absence of the keyword ArtScience or SciArt, since STEAM is an acronym that does not represent all emerging literature on the arts as an entry point to science evidence.*

**Response of the authors:** Thank you for this important point. We have now added ArtScience and SciArt as potential terms for the search strategy in addition to STEAM in a new Table 3 and a revised search strategy in Table 4.

*Independently of these concerns, my overall opinion on the paper is that it deserves publication due to the originality and relevance of the subject, and adequacy of methodology.*

**Response of the authors:** Thank you for the positive feedback on our paper.

**Response to academic editor**

Journal Requirements:

*When submitting your revision, we need you to address these additional requirements.*

1. *Please ensure that your manuscript meets PLOS ONE's style requirements, including those for file naming. The PLOS ONE style templates can be found at*
   [*https://journals.plos.org/plosone/s/file?id=wjVg/PLOSOne_formatting_sample_main_body.pdf*](https://journals.plos.org/plosone/s/file?id=wjVg/PLOSOne_formatting_sample_main_body.pdf) *and* 
   [*https://journals.plos.org/plosone/s/file?id=ba62/PLOSOne_formatting_sample_title_authors_affiliations.pdf*](https://journals.plos.org/plosone/s/file?id=ba62/PLOSOne_formatting_sample_title_authors_affiliations.pdf)

**Response of the authors:** we have checked that the style of our manuscript follows the guidelines and used the requested file names.

*2. Thank you for stating in your Funding Statement: 
"This work has been supported by the Irish Research Council* [*https://research.ie/*](https://research.ie/) *under grant number COALESCE/2022/1664 (HP, AH)."*

*Please provide an amended statement that declares *all* the funding or sources of support (whether external or internal to your organization) received during this study, as detailed online in our guide for authors at* [*http://journals.plos.org/plosone/s/submit-now*](http://journals.plos.org/plosone/s/submit-now)*.  Please also include the statement “There was no additional external funding received for this study.” in your updated Funding Statement. 
Please include your amended Funding Statement within your cover letter. We will change the online submission form on your behalf.*

**Response of the authors:** we have amended the funding statement to "This work has been supported by the Irish Research Council <https://research.ie/> under grant number COALESCE/2022/1664 (HP, AH). There was no additional external funding received for this study”.

*3. Please include captions for your Supporting Information files at the end of your manuscript, and update any in-text citations to match accordingly. Please see our Supporting Information guidelines for more information:* [*http://journals.plos.org/plosone/s/supporting-information*](http://journals.plos.org/plosone/s/supporting-information)*.*

**Response of the authors:** We have included a caption for the supporting information file at the end of our manuscript and updated the in-text citation.

*4. Please review your reference list to ensure that it is complete and correct. If you have cited papers that have been retracted, please include the rationale for doing so in the manuscript text, or remove these references and replace them with relevant current references. Any changes to the reference list should be mentioned in the rebuttal letter that accompanies your revised manuscript. If you need to cite a retracted article, indicate the article’s retracted status in the References list and also include a citation and full reference for the retraction notice.*

**Response of the authors:** We have checked our reference list. There are no changes.
